# Micro Ethanol Sensors with a Heater Fabricated Using the Commercial 0.18 μm CMOS Process

**DOI:** 10.3390/s131012760

**Published:** 2013-09-25

**Authors:** Wei-Zhen Liao, Ching-Liang Dai, Ming-Zhi Yang

**Affiliations:** Department of Mechanical Engineering, National Chung Hsing University, Taichung 402, Taiwan; E-Mails: caca_78125@yahoo.com.tw (W.-Z.L.); d099061005@mail.nchu.edu.tw (M.-Z.Y.)

**Keywords:** ethanol sensor, zinc oxide film, heater, post-process

## Abstract

The study investigates the fabrication and characterization of an ethanol microsensor equipped with a heater. The ethanol sensor is manufactured using the commercial 0.18 μm complementary metal oxide semiconductor (CMOS) process. The sensor consists of a sensitive film, a heater and interdigitated electrodes. The sensitive film is zinc oxide prepared by the sol-gel method, and it is coated on the interdigitated electrodes. The heater is located under the interdigitated electrodes, and it is used to supply a working temperature to the sensitive film. The sensor needs a post-processing step to remove the sacrificial oxide layer, and to coat zinc oxide on the interdigitated electrodes. When the sensitive film senses ethanol gas, the resistance of the sensor generates a change. An inverting amplifier circuit is utilized to convert the resistance variation of the sensor into the output voltage. Experiments show that the sensitivity of the ethanol sensor is 0.35 mV/ppm.

## 1. Introduction

Ethanol sensors are important devices for application in industrial and environmental monitoring. Humans may inhale high concentrations of ethanol vapor that can cause headaches, balance disorders, nausea, dizziness and confusion [1]. In addition, human skin contact with ethanol vapor may result in eye and mucous membrane irritation, dissolved skin oils and interference with physiological functions [1]. Therefore, ethanol sensors play an important role in preventing the damages caused by ethanol.

The advantages of microsensors include small size, low cost, high performance and easy mass-production. Micromachining technology was used to fabricate various microsensors [2]. Many traditional gas sensors were miniaturized as gas microsensors using this technology. For instance, Peng, *et al.* [3] employed micromachining technology to make a nitrogen oxide microsensor, whose sensitive material was porous silicon nanowires prepared by the metal-assisted chemical etching method. The gas microsensor had an ability to sense nitrogen oxide at room temperature. Joshi and Kumar [4] reported room temperature gas microsensors based on silicon nanowires manufactured by micromachining technology. Dong *et al.* [5] proposed a gas microsensor with a micro heater fabricated using micromachining technology. The sensitive material of the gas sensor was Pt doped SnO_2_ nanofibers. The micro heater provided a working temperature to the sensor. The sensor was used to detect H_2_S gas. Kliche *et al.* [6] used microelectromechanical system (MEMS) technology to manufacture a sensor for the thermal detection of changes of gas mixtures such as the CO_2_ concentration in air. The sensor had three silicon microwires surrounded by the gas mixture to be analyzed. A centered wire was supplied with sinusoidal heating power. Liu *et al.* [7] developed a micro catalytic combustible hydrogen gas sensor using MEMS technology, and the sensitive material of the sensor was tin dioxide. A chemical vapor deposition method was utilized to deposit a porous nano-crystalline SnO_2_ layer on the sensor.

Several ethanol microsensors have been fabricated using micromachining technology. For instance, Chen *et al.* [8] developed a back-gated graphene field effect transistor (FET) array on microchannels for sensing ethanol vapor. The sensing material of the sensor was graphene. Microchannels fabricated on a SU-8 (a negative photoresist) substrate with top metal electrodes were pressed onto another silicon/SiO_2_ substrate with predeposited graphene pieces. Pandya *et al.* [9] employed MEMS technology to make an ethanol sensor, and a nickel micro heater was integrated with the sensor. The sensing film of the sensor was ZnO nanostructures. The sensor was sensitive and selective to ethanol vapor at an operating temperature of 100 °C. The resistance of the sensor produced a change when it sensed ethanol vapor. Shin *et al.* [10] presented a piezoelectrically driven microbridge ethanol sensor fabricated by MEMS process. The sensing layer of the sensor was PMMA polymer. The resonant frequency of the microbridge transducer changed upon the mass of sensing layer increased. The sensor had the sensitivity of 1.67 ppm/Hz for ethanol vapor. Their sensors [8–10] were not integrated with circuitry.

Recently, the commercial CMOS process was employed to develop various micro devices [11–15]. Micro devices manufactured by this process have a potential for integration with circuits on-a-chip [16–18]. In this study, an ethanol sensor with a heater is fabricated using the CMOS process and connected with an inverting amplifier circuit. Zinc oxide is adopted as the sensitive material of the sensor since it has a high sensitivity and a high selectivity for ethanol gas [19]. The ethanol sensor requires a post-process to coat the sensitive material. The post-process contains to remove the sacrificial oxide layer and coat the zinc oxide film.

## 2. Structure of the Ethanol Sensor

**Figure 1.**
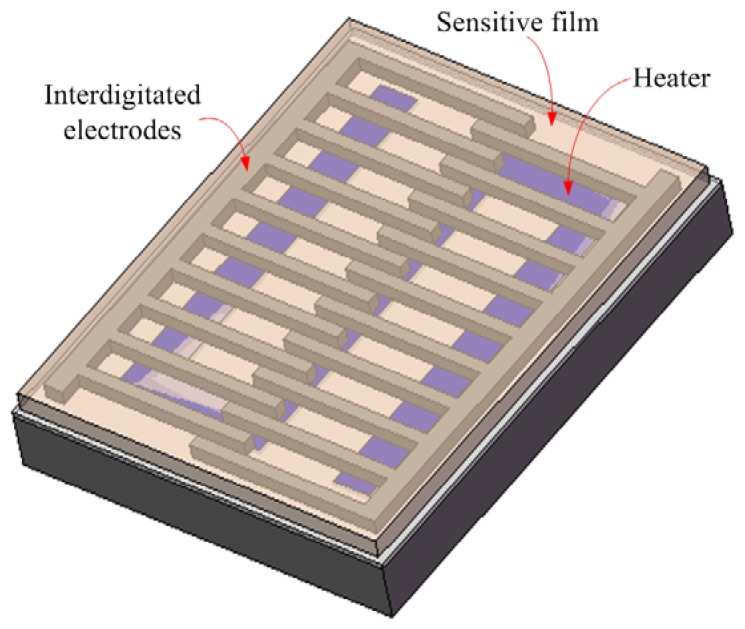
Schematic structure of the ethanol sensor.

Figure 1 illustrates schematic structure of the ethanol sensor chip. The sensor chip is made up of a heater, a sensitive film and interdigitated electrodes. The heater is located under the interdigitated electrodes, and it is used to provide the working temperature. Material of the heater is polysilicon, and the heater is designed as a winding line, in which the dimensions are 2,200 μm long, 35 μm wide and 0.9 μm thick. The interdigitated electrodes are formed by aluminum and tungsten metals, and their dimensions are 200 μm long, 20 μm wide and 6 μm thick, and the gap between the electrodes is 40 μm. Area of the sensor chip is about 0.3 mm^2^. The sensitive material of the sensor is zinc oxide, and it is coated on the interdigitated electrodes. The reaction mechanism of zinc oxide adsorbing ethanol gas is given by [19]:

(1) $$\frac{1}{2}O_{2}+2e^{-}\to 2O^{-}$$

and:

(2) $$CH_{3}CH_{2}OH+6O^{-}\to 2CO_{2}+3H_{2}O+e^{-}$$

where *O*^−^ denotes the oxygen ion on the surface of zinc oxide film and *e*^−^ is a conduction electron. According to Equations (1) and (2), when the zinc oxide film adsorbs ethanol gas, the mobility of electrons in the sensitive film increases because the electrons in the film increase, resulting in the resistance of the film decreases. Thereby, the ethanol sensor is a resistive type. When the sensitive film adsorbs ethanol gas, the gas reacts with negative oxygen ions on the surface of zinc oxide, leading to the resistance of the ethanol sensor decreases. Oppositely, the resistance of the ethanol sensor increases when the sensitive film desorbs ethanol gas.

**Figure 2.**
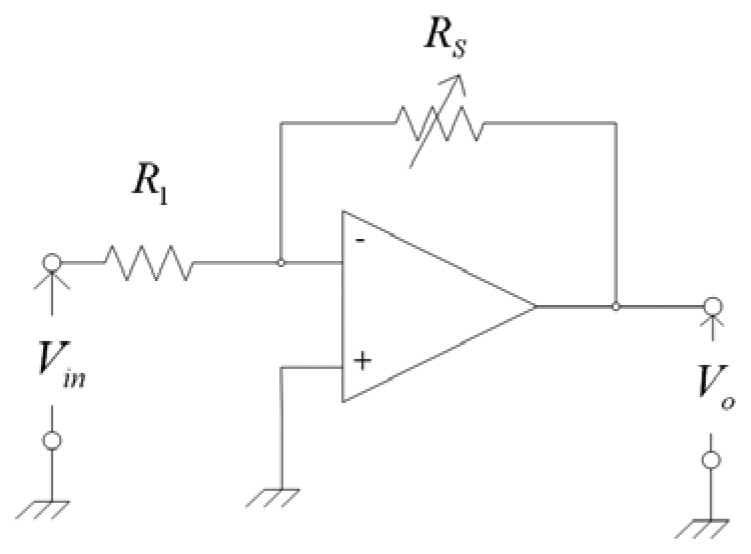
Inverting amplifier circuit for the ethanol sensor.

**Figure 3.**
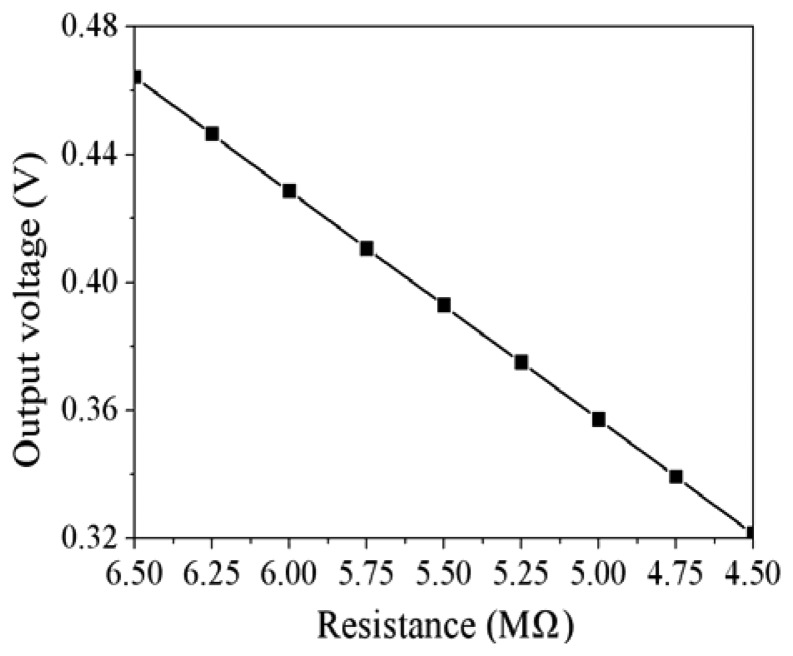
Evaluated results of the output voltage for the circuit.

In order to obtain the output voltage of the ethanol sensor, an inverting amplifier circuit is connected with the ethanol sensor. Figure 2 illustrates the inverting amplifier circuit for the ethanol sensor [20]. The inverting amplifier circuit is employed to convert the resistance variation of the ethanol sensor into the output voltage. The output voltage of the inverting amplifier circuit is given by [20]:

(3) $$\left|V_{0}\right|=\left|-\frac{R_{s}}{R_{1}}V_{in}\right|$$

where *V_o_* represents the output voltage of the circuit; *V_in_* is the input voltages of the circuit; *R_s_* is the resistance of the ethanol sensor; *R_1_* is the resistance of the circuit. According to Equation (3), the output voltage of the ethanol sensor can be obtained. Figure 3 shows the evaluated results of the circuit for the ethanol sensor. In this investigation, the resistance of R_1_ is 1.4 MΩ, and the input voltages of *V_in_* is 0.1 V. The resistance of the ethanol sensor changes from 6.5 to 4.5 MΩ. The evaluated results show that the output voltage of the circuit decrease from 460 to 320 mV as the resistance of the sensor changes from 6.5 to 4.5 MΩ.

## 3. Preparation of the Sensitive Film

The sensitive film of the ethanol sensor was zinc oxide prepared by sol-gel method [19]. The zinc oxide was prepared as follows: zinc acetate dehydrate (3 g) was dissolved in isopropyl alcohol (100 mL) with stirring for 10 min until the solution was uniformly mixed. The mixed solution was mixed with ethanolamine (4 g) and stirred for 1 h at 50 °C, followed by aging the mixed solution for 24 h. After the reaction, the resulting products were filtered, and washed with isopropyl alcohol and deionized water. Finally, the zinc oxide was coated on the substrate, followed by calcination at 350 °C for 3 h.

**Figure 4.**
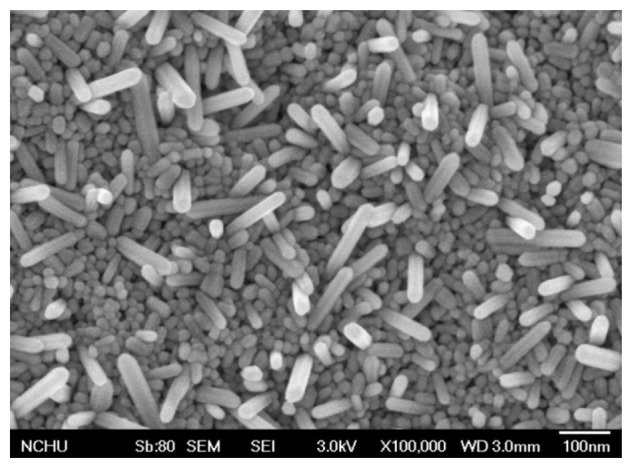
SEM image of the zinc oxide film.

**Figure 5.**
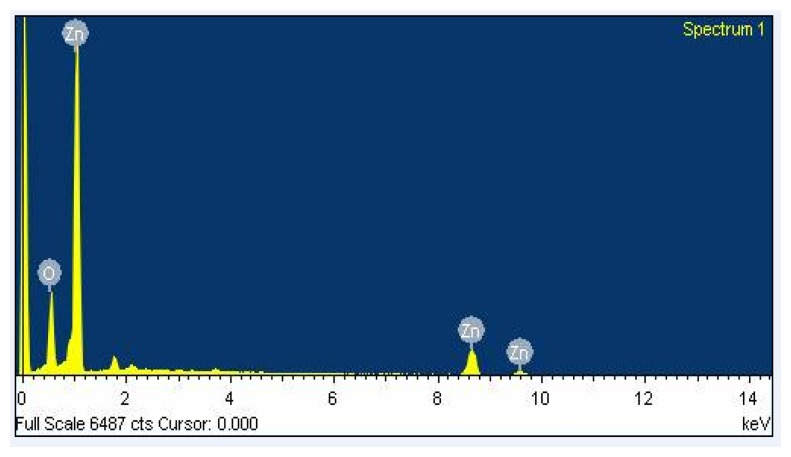
EDS analysis of zinc oxide.

The surface morphology of the zinc oxide film was measured by scanning electron microscopy (SEM). Figure 4 shows a SEM image of the zinc oxide film. The film has a nanorod structure that has a large surface area that helps to increase its sensitivity. The composition of the zinc oxide film was tested by an energy dispersive spectrometer (EDS). Figure 5 shows an EDS analysis of zinc oxide. The measured results revealed that the film contained zinc of 77.71 wt% and oxygen of 22.29 wt%.

## 4. Fabrication of the Ethanol Sensor

**Figure 6.**
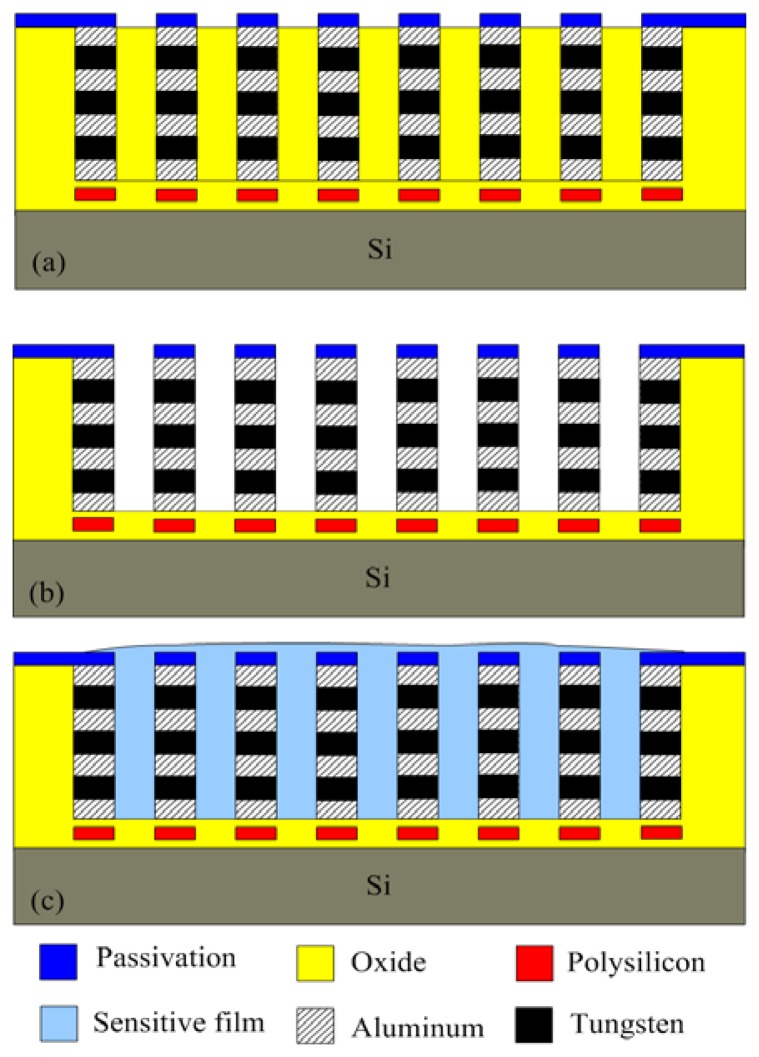
Fabrication process of the ethanol sensor: (**a**) after the CMOS process; (**b**) etching the sacrificial layer; (**c**) coating the sensitive film.

The commercial 0.18 μm CMOS process of Taiwan Semiconductor Manufacturing Company (TSMC, Taipei, Taiwan) was employed to fabricate the ethanol sensor. Figure 6 displays the fabrication flow of the ethanol sensor. After completion of the CMOS process [21], the ethanol sensor needed a post-processing step to coat the zinc oxide film.

**Figure 7.**
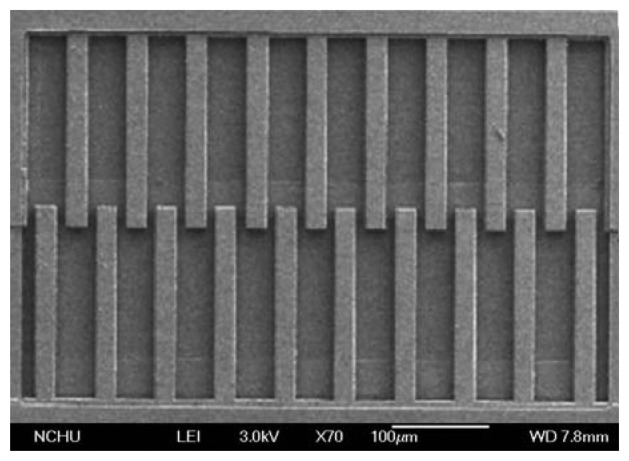
SEM image of the ethanol sensor after the wet etching.

**Figure 8.**
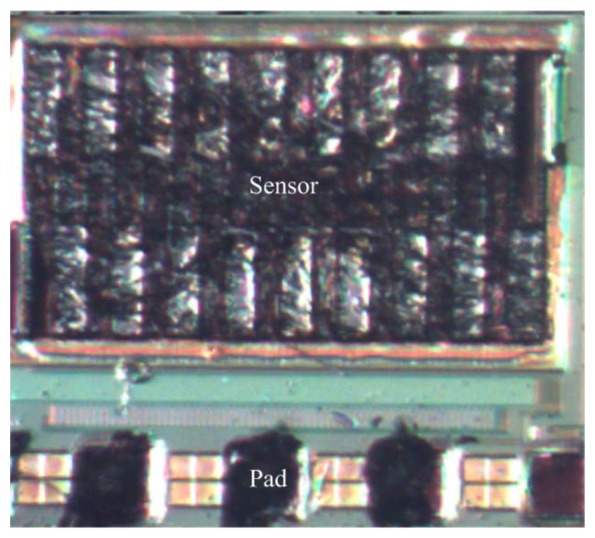
Optical image of the ethanol sensor after the post-processing step.

The post-process consisted of two steps: (1) the sacrificial oxide layer between the interdigitated electrodes was removed; (2) the zinc oxide was coated on the interdigitated electrodes. Figure 6a illustrates the cross-section of the ethanol sensor after the CMOS process. The sacrificial oxide layer between the interdigitated electrodes required to remove. Figure 6b shows that the sacrificial oxide layer is removed. A wet etching with BOE (buffer oxide etch) solution was used to etch the sacrificial oxide layer [22–24], and to expose the interdigitated electrodes. The interdigitated electrodes were formed by a stack of aluminum and tungsten metals. Figure 6c presents that the sensitive film is coated. The zinc oxide was dropped on the interdigitated electrodes using a precision-control micro-dropper, followed by the zinc oxide film was calcinated at 350 °C for 3 h. Figure 7 shows a SEM image of the interdigitated electrodes after the wet etching. Figure 8 shows an optical image of the ethanol sensor after the post-processing step.

## 5. Results and Discussion

**Figure 9.**
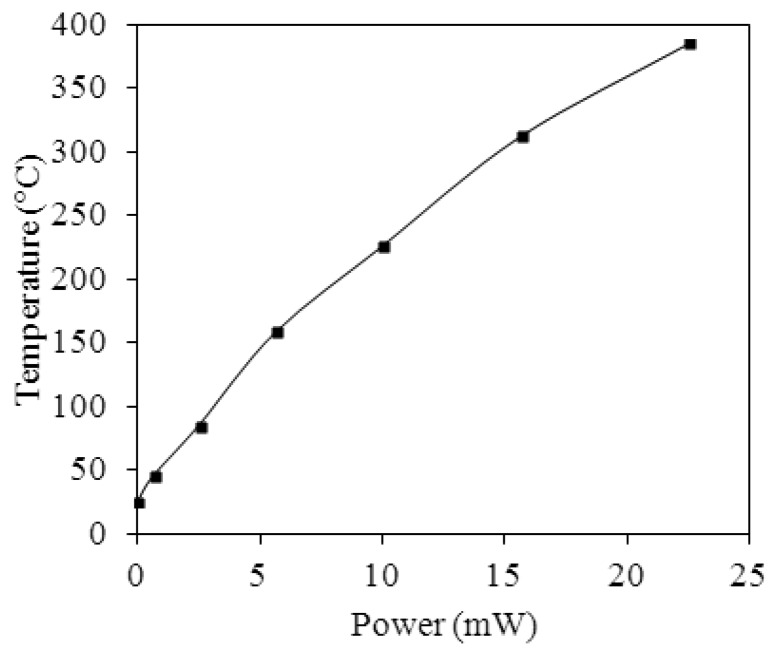
Measured results of the heater.

A power supply, an infrared thermometer and a LCR meter were used to measure the characteristics of the heater in the ethanol sensor. The power supply provided a power to the heater, and the infrared thermometer detected the temperature of the heater. Figure 9 presents the measured result of the heater. The results showed that the heater generated a temperature of 350 °C when applying a power of 19.5 mW to it.

**Figure 10.**
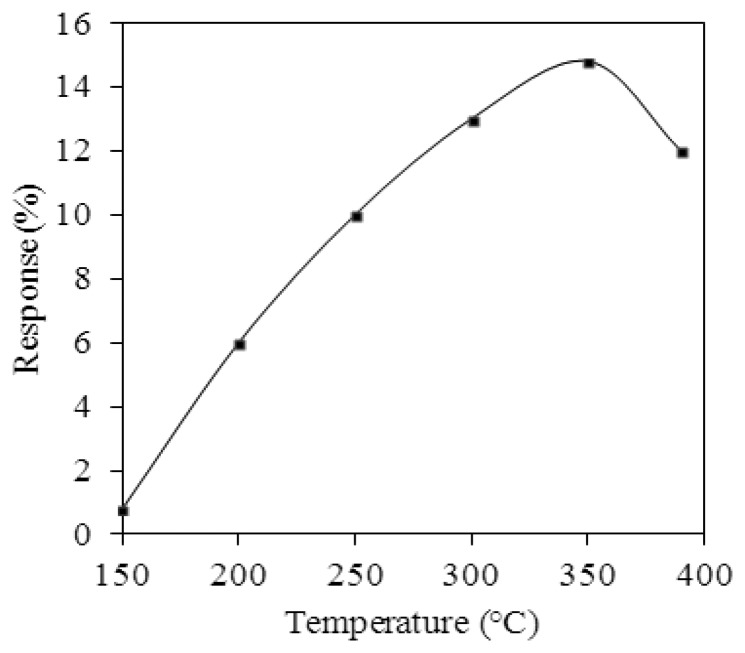
Response of the ethanol sensor at 150 ppm ethanol.

The performance of the ethanol sensor was tested by a test chamber, a power supply, a LCR meter and an oscilloscope. The test chamber contained a calibration ethanol sensor (BW GasAlertMicro5 PID, Honeywell Taiwan Ltd., Taipei, Taiwan), a control valve, and a pump. The calibration ethanol sensor was set in the test chamber for monitoring *in-situ* the ethanol concentration in the test chamber. The ethanol concentration was tuned by the control valve. The pump was used to exhaust the ethanol gas in the test chamber upon finishing the testing. The ethanol sensor was tested under different temperatures in order to characterize its best working temperature. The ethanol sensor was set in the test chamber, and the control valve allowed ethanol gas to enter the test chamber. The control valve was closed when the calibration ethanol sensor displayed a concentration of 150 ppm, and the concentration of the test chamber was maintained constant. The heater provided different working temperatures to the ethanol sensor, and the LCR meter detected the resistance of the sensor. Figure 10 displays the response of the ethanol sensor at 150 ppm ethanol. The response is defined as:

(4) $$\left|\frac{R_{s}-R_{o}}{R_{o}}\right|\times 100\%$$

where *R_o_* represents the initial resistance of the ethanol sensor and *R_s_* is the resistance variation of the ethanol sensor. The measured results revealed that the best working temperature for the ethanol sensor was 350 °C.

As shown in Figure 10, the best working temperature of the ethanol sensor was 350 °C. The ethanol sensor was measured under different concentrations at 350 °C, and the LCR meter was used to record the resistance variation of the sensor.

**Figure 11.**
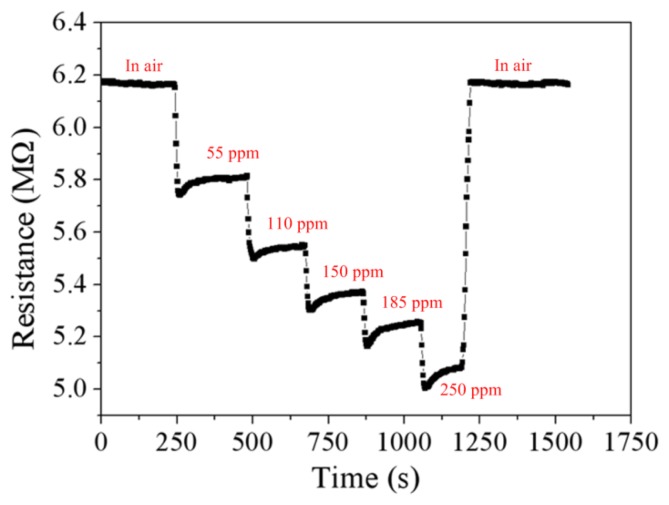
Test of the ethanol sensor.

**Figure 12.**
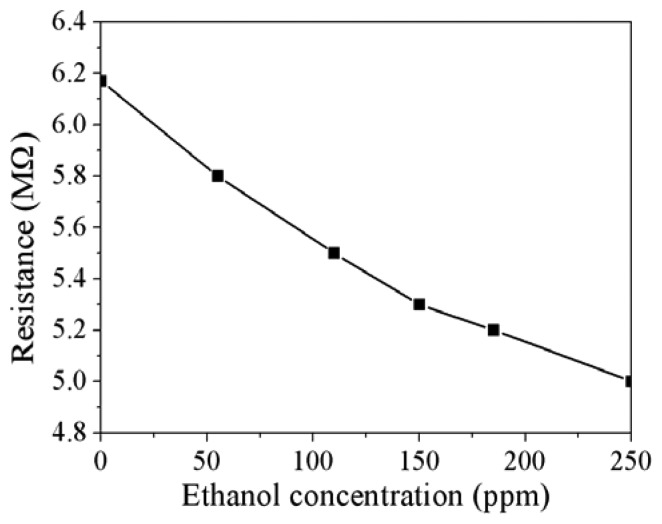
Resistance variation of the ethanol sensor.

Figure 11 demonstrates the resistance variation of the ethanol sensor at different ethanol concentrations. The measured results showed that the initial resistance of the ethanol sensor was 6.18 MΩ in air, and the resistance of the sensor reduced to 5.1 MΩ at 250 ppm ethanol. The sensor recovered to the initial resistance of 6.18 MΩ when it was in air. Figure 12 shows the relation between the resistance and ethanol concentration for the ethanol sensor. The resistance of the ethanol sensor decreased as the concentration of ethanol increased. The inverting amplifier circuit was employed to convert the resistance of the ethanol sensor into the output voltage. The ethanol sensor connected with the circuit was set in the test chamber, and the heater provided a working temperature of 350 °C to the sensor. The power supply provided a bias voltage of 5 V and an input voltage of 0.1 V to the circuit. The ethanol sensor with the circuit was tested under different ethanol concentrations, and the output voltage of the circuit was detected by the oscilloscope.

**Figure 13.**
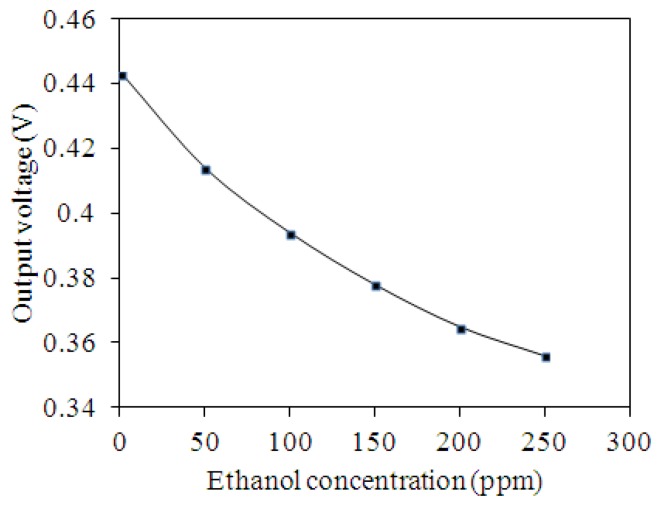
Measured results of the output voltage for the ethanol sensor with the circuit.

Figure 13 depicts the output voltage of the ethanol sensor with the inverting amplifier circuit. The measured results showed that the output voltage of the ethanol sensor changed from 443 to 356 mV as the concentration of ethanol gas increased from 0 to 250 ppm.

The variation of the output voltage was 87 mV in 0–250 ppm ethanol. Therefore, the sensitivity of the ethanol sensor was about 0.35 mV/ppm.

## 6. Conclusions

An ethanol sensor with a heater has been fabricated using the commercial 0.18 μm CMOS process. The sensitive material of the ethanol sensor was zinc oxide prepared by sol-gel method. The sensor required a post-processing step to etch the sacrificial oxide layer and coat the sensitive material. In the post-processing, wet etching was used to remove the sacrificial oxide layer between the interdigitated electrodes, followed by coating the zinc oxide film on the interdigitated electrodes. Experiments showed that the best working temperature of the sensitive zinc oxide film was 350 °C. The heater, which was located under the interdigitated electrodes, provided a working temperature of 350 °C to the sensitive film. When the sensitive film absorbed or desorbed ethanol gas, the ethanol sensor produced a change in resistance. An inverting amplifier circuit was adopted to convert the resistance variation of the sensor into the output voltage. The experimental results showed that the resistance of the ethanol sensor varied from 6.18 to 5.1 MΩ as the ethanol concentration increased from 0 to 250 ppm at 350 °C. The sensitivity of the ethanol sensor with the inverting amplifier circuit was 0.35 mV/ppm.
